# Sustainable Smart Packaging from Protein Nanofibrils

**DOI:** 10.1002/adma.202414658

**Published:** 2024-11-20

**Authors:** Mohammad Peydayesh, Alan Kovacevic, Leah Hoffmann, Felix Donat, Ciatta Wobill, Laura Baraldi, Jiangtao Zhou, Christoph R. Müller, Raffaele Mezzenga

**Affiliations:** ^1^ Department of Health Sciences and Technology ETH Zurich Zurich 8092 Switzerland; ^2^ Department of Mechanical and Process Engineering ETH Zürich Leonhardstrasse 21 Zürich CH‐8092 Switzerland; ^3^ Department of Materials ETH Zurich Zurich 8093 Switzerland

**Keywords:** amyloid fibrils, bioplastics, food sidestreams, protein nanofibrils, smart packaging

## Abstract

Smart packaging technologies are revolutionizing the food industry by extending shelf life and enhancing quality monitoring through environmental responsiveness. Here, a novel smart packaging concept is presented, based on amyloid fibrils (AM) and red radish anthocyanins (RRA), to effectively monitor food spoilage by color change. A protein nanofibrils biofilm is developed from whey protein, which is functionalized with RRA to endow the resulting films with advanced monitoring capabilities. A comprehensive characterization, including pH responsiveness, water vapor permeability, thermal and mechanical testing, and colorimetric responses, demonstrates the superiority of AM/RRA films compared to control films based on whey monomer building blocks. The findings indicate that the AM/RRA films can effectively monitor, for example, shrimp freshness, showing visible changes within one day at room temperature and significant alterations in color after two days. Furthermore, these films exhibit high antibacterial and antioxidant activities, reinforcing their suitability for efficient food packaging. By integrating bio‐based materials from whey and natural anthocyanins, this research presents a biodegradable, sustainable, and cost‐effective smart packaging solution, contributing to eco‐friendly innovations in food preservation.

## Graphical abstract

Smart packaging can extend shelf life and improve food quality monitoring. This study introduces a novel smart packaging solution using amyloid fibrils and red radish anthocyanins to monitor seafood spoilage. These findings show that these films effectively indicate shrimp freshness and possess high antibacterial and antioxidant properties, offering a sustainable approach to food packaging.

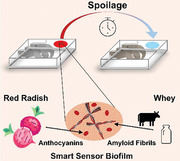

## 1 Introduction

Food waste is a significant global issue, with approximately one‐third of all food produced being lost or discarded annually, impacting massively food security and environmental sustainability.^[^ ^1^ ^]^ Valorizing side‐streams from the agriculture and food industry offers a promising strategy to tackle this challenge.^[^ ^2^, ^3^ ^]^ In the dairy sector, whey–a by‐product of cheese production–accounts for 85–95% of milk volume and contains ∼20% of its protein content.^[^ ^4^ ^]^ Recent advancements have demonstrated that β‐lactoglobulin, the major protein in whey, can be converted into amyloid fibrils (AM), providing a platform for developing biodegradable plastics with superior mechanical properties.^[^ ^5^ ^]^ AM, formed under conditions of low pH and elevated temperatures, consists of highly ordered β‐sheet aggregates that endow remarkable structural robustness to plastic films.^[^ ^6^, ^7^ ^]^

The role of food packaging in ensuring the safety and preservation of perishable products is of paramount importance.^[^ ^8^ ^]^ However, conventional plastic packaging, often non‐biodegradable, contributes significantly to environmental problems, including plastic pollution, microplastic contamination, and the use of toxic chemicals in their production. This underscores the urgent need for biodegradable and sustainable packaging solutions.^[^ ^9^ ^]^ Beyond sustainability, smart packaging technologies have emerged, designed to either prolong shelf life or monitor food quality by responding to inside environmental changes.^[^ ^10^ ^]^ For instance, smart packaging that detects spoilage through pH changes caused by volatile basic nitrogen (VB‐N) compounds–such as ammonia and trimethylamine, which are produced during seafood decomposition–has gained considerable interest.^[^ ^11^, ^12^, ^13^ ^]^ This is especially relevant for highly perishable products such as shrimp, where sensitive detection systems can help reduce both food waste and food‐borne illnesses.^[^ ^14^, ^15^ ^]^

Many existing pH‐responsive biofilms incorporate synthetic, petroleum‐based dyes, which can be non‐biodegradable and potentially harmful if ingested. In contrast, natural plant‐based dyes are non‐toxic, abundant, and sustainable alternatives.^[^ ^16^, ^17^ ^]^ Among these phytochemicals are anthocyanins, which are water‐soluble compounds found in, e.g., red radishes (RRA), that have a broad range of color.^[^ ^18^, ^19^ ^]^ Anthocyanins are hydrophilic molecules with a flavonoid backbone.^[^ ^20^ ^]^ Anthocyanins are constituted by many compounds with different pK_a_ and charged states. The backbone is the same for every compound, but the substitution of the rings (H, OH, CH_3_) differ.^[^ ^21^ ^]^ The main components of anthocyanins in red radishes are pelargonidin‐3‐sophoroside‐5‐glucoside (P), glucoside‐ethylidene‐catechin, and pelargonidin‐(feruloyl) diglucoside‐5‐(malonyl) glucoside‐ethylidene‐catechin.^[^ ^22^ ^]^

Anthocyanins have been widely studied for use in smart films designed to monitor the freshness of perishable foods such as seafood and meat.^[^ ^23^, ^24^ ^]^ In particular, their colorimetric response to pH changes provides a clear visual indication of spoilage, with several studies demonstrating their effectiveness in detecting food freshness by integrating them into biodegradable films.^[^ ^25^, ^26^ ^]^ For example, one study developed a film incorporating anthocyanins from black rice to monitor the freshness of seafood,^[^ ^25^ ^]^ while another utilized anthocyanins extracted from Roselle for similar purposes in pork packaging.^[^ ^26^ ^]^ Yet, some limitations regarding the applications of anthocyanins have been reported, including color instability under certain conditions^[^ ^27^ ^]^ and weaker mechanical properties of the films.^[^ ^28^ ^]^

In this work, we present a novel smart bioplastic packaging combining AM derived from whey protein with anthocyanins extracted from red radishes. Both components remain stable at low pH (≈2), facilitating their integration into a unified system.^[^ ^29^ ^]^ Thanks to this hybridization, our film exhibits enhanced color stability, antimicrobial and antioxidative capacities, and superior mechanical strength compared to previous studies.^[^ ^30^, ^31^ ^]^ The AM structural integrity not only improves the physical properties of the bioplastic but also enables precise, sensitive colorimetric detection of seafood spoilage, providing an efficient and sustainable solution for monitoring food freshness and extending shelf life.

## 2 Results and Discussion

### 2.1 AF Smart Packaging Preparations

**Figure 1:**
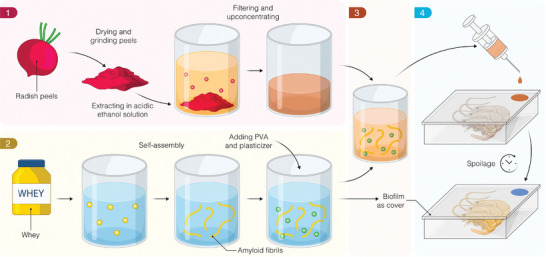
Schematic of the process followed for the fabrication of dye extract from radish peels, the biofilm based on whey nanofibrils, and its functionalization into smart packaging films.

The process for producing the smart packaging (**Figure** **1**) starts by extracting the RRA pigments in an acidic ethanol solution (step 1) and transforming whey protein into AM (step 2). These nanofibers are subsequently combined with polyvinyl alcohol (PVA) and glycerol as a plasticizer to produce biofilms by solvent casting. This biofilm is eventually functionalized with RRA to achieve its monitoring capabilities (step 3). The last step is the application of the AM/RRA sensor films for shrimp spoilage (step 4) on top of the hybrid AM packaging film.

### 2.2 pH‐Response of RRA and Its Hybridization of AM

**Figure 2:**
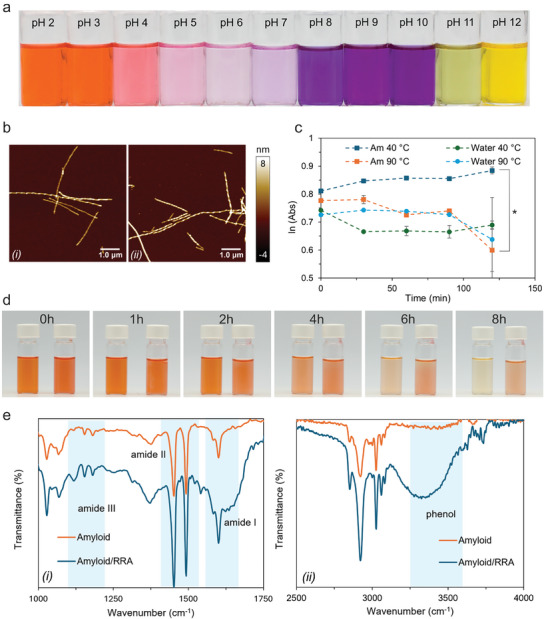
a) pH‐series of RRA extract (the extract had a dry mass of 62 ± 1 wt.%, thus a concentration of 1 mg mL^−1^, or 1 g L^−1^ has a dry mass of 620 mg L^−1^); b) AFM of pure AM (i), and AM with anthocyanin (ii); c) Thermal degradation of RRA in water *vs* in AM solution (both at pH 2) at 40 and 90 °C (data are represented as means ± SD, where *n* = 3 and ^*^ *p* < 0.05); d) RRA degradation in water (left) *vs* in AM solution of 2 wt.% (right) both at pH 2, e) FT‐IR of AM *vs* AM/RRA (AM specific region (i) and phenol specific region (ii)).

The pH responses of RRA solutions are presented in **Figure** **2**a. The extracted RRA exhibited distinct color changes across a pH range of 2 to 12, easily detectable by the naked eye. These colors transitioned from red to translucent, blue/violet, green, and yellow. As a natural colorant, RRA is an excellent match for combining AM and PVA‐based bioplastic to create a pH‐sensitive sensor for intelligent food packaging. Its ability to produce multiple distinct color changes across the acidic to basic spectrum, along with its simple extraction process and stable properties, makes it ideal for such applications. Atomic force microscopy (AFM) images in Figure 2b further confirmed the structural integrity of the AM after dye incorporation, whereby no visible differences were observed between the AM alone (i) and the AM hybridized with RRA (ii).

To assess the stability of the dye‐protein solutions, we compared them to the dye alone in water (Figure 2c,d). Up to 40 °C, there was no thermal degradation of the anthocyanins, and samples containing AM demonstrated significantly better anthocyanins preservation than the aqueous dye solution. Anthocyanins are most stable at low pH, such as pH 2, which aligns with the conditions for AM preparation and film casting.^[^ ^32^ ^]^ This enhanced stability is evident in Figure 2d, where the AM‐containing samples retained their color more effectively than the water‐based samples after 8 h. However, at higher temperatures, anthocyanin degradation occurs, and no significant difference was observed between the AM‐containing and aqueous samples.

The Fourier Transformed Infrared (FT‐IR) spectra in Figure 2e‐i confirmed the formation of AM in both samples, as all three AM‐specific amide bands were present, though a difference in absorbance is noted.^[^ ^20^ ^]^ Moreover, Figure 2e‐ii indicates the presence of phenolic moieties coming from the added RRA (broad band ≈3300 cm^−1^).^[^ ^20^ ^]^ All the other peaks overlap perfectly comparing the two samples; thus, the impact of RRA on the properties of the AM is likely to be minimal. This is probably due to the relatively small size of flavonoid molecules compared to the much larger protein and AM aggregates. Additionally, the amount of RRA (0.1 wt.%) used in the AM (4 wt.%) preparation is very low.

### 2.3 Characterization of the Biofilm

**Figure 3:**
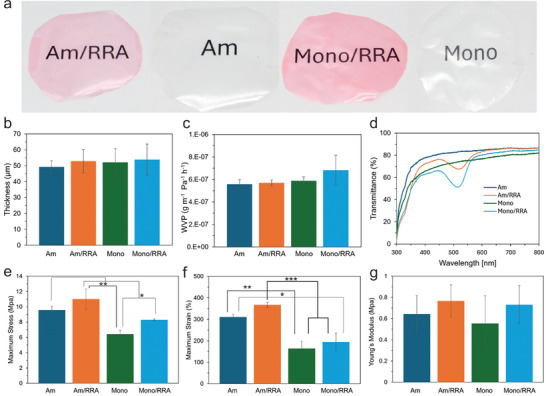
a) Visual appearance of biofilms; b) Thickness of films (data are represented as means ±  SD, where *n* = 8); c) Water vapor permeability (data are represented as means ± SD, where *n* = 4); d) Light transmittance by UV–vis of films. Mechanical properties: e) Maximum stress (data are represented as means ± SD, where *n* = 3, ^*^ *p* < 0.05, ^**^ *p* < 0.005); f) Maximum strain(data are represented as means ± SD, where *n* = 3, ^*^ *p* < 0.05, ^**^ *p* < 0.005, ^***^ *p* < 0.001); g) Young's modulus of biofilms (data are represented as means ± SD, where *n* = 3).

**Figure** **3**a shows the visual appearance of the protein‐based sensors incorporating RRA. As observed, the films containing RRA, specifically both the hybrid bioplastics made from AM (AM/RRA) and monomers (Mono/RRA), exhibited a distinct red color. This is in contrast to the pure hybrid biofilms using proteins without dye in both AM and native monomer (Mono) forms, which were colorless and transparent. Furthermore, the AM‐based samples had greater transparency compared to the monomer‐based ones.

The thickness of all films was homogeneous, measuring ≈52 ± 1.7 µm, as shown in Figure 3b. This was expected since the main components and their ratios, including the viscosity of both solutions, were the same. The addition of only 0.1 wt.% RRA did not significantly impact the film thickness.

The results concerning water vapor permeability, shown in Figure 3c, revealed a significant difference between the Mono/RRA film and the other three samples. Mono/RRA exhibits the highest permeability of water vapor, making it less suitable as a packaging material. This may be attributed to the hydrophilic nature of both whey monomers^[^ ^5^ ^]^ and anthocyanin, which together amplify this effect. The light transmittance measurements in Figure 3d indicate lower transmittance of both colored films at ≈500 nm. However, the overall trend remains consistent, with a slight shift toward the left attributed to the opaque nature of whey, which contributed to the reduced transmittance in the Mono/RRA film.

The release kinetics of soluble components, shown in Figure S1 (Supporting Information), demonstrated similarly consistent behavior across the films. However, the Mono film had a much faster initial release within the first hour, more than double that of the other films. This difference was likely because the monomers did not form a network in the film, unlike the AM, making them more easily dissolved in water. This may also explain why the RRA was released more rapidly from the Mono film. The thermal gravimetric analysis (TGA) of all four films, shown in Figures S2 and S3 (Supporting Information), revealed no significant differences, most likely because the primary components of the films remained the same across all samples.

The mechanical tests (Figure S4, Supporting Information) demonstrate that the AM/RRA film had superior physical properties, including maximum stress, maximum strain, and Young's modulus, compared to the other films (Figure 3e–g). This trend was also observed in the Mono films, where Mono/RRA showed better properties than the pure Mono film. For maximum stress and maximum strain, the differences between films with and without RRA were comparable (Figure 3e,f). Notably, Young's modulus measurements (Figure 3g) revealed that both films containing RRA were more elastic, with Mono/RRA even surpassing the Am film. These results suggest that AM films combined with RRA offer enhanced physical and chemical properties, making them ideal candidates for smart sensor applications. The improved mechanical properties may be attributed to hydrophobic interactions between RRA and the AM, as well as the branched structure and increased cross‐linking from the sugar residues of pelargonidin.^[^ ^30^, ^31^ ^]^

### 2.4 pH‐Sensitive Smart Sensor

**Figure 4:**
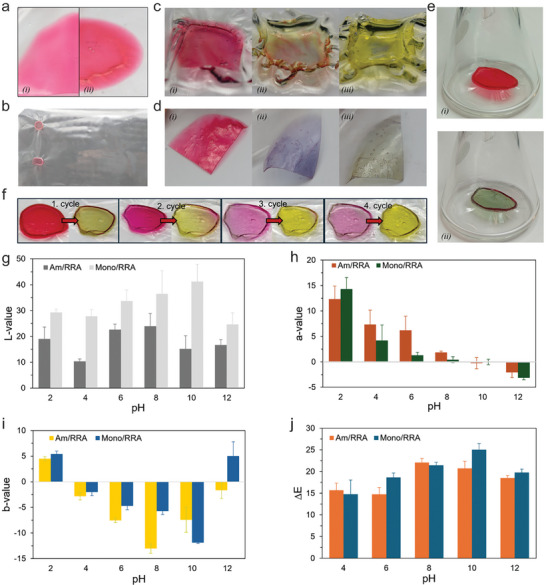
a) Smart sensor with colored added before drying (i) and after drying (ii); b) Smart sensor attached to larger biofilm; c) Smart sensor exposed to 0.1 M NaOH and subsequent change in color at the beginning (i), after 5 min (ii) and 10 min (iii); d) response to different vapor compositions: pH 2 acetic acid (i), pH 10 1:10 NH_3_ (ii), pH 12 NH_3_ (iii); e) reaction to ammonia gas before (i) and after (ii) exposure; f) Reversibility in pH‐change of smart sensor from vapor over 4 cycles; Changes in color values of the monomer‐ and AM‐based films at various pH‐values in g) Brightness; h) Redness; i) Yellowness and j) Overall change (all data are represented as means ± SD, where *n* = 3).

For application in food packaging, the pH‐sensitive film is designed to be circular and attached to a larger bioplastic film (**Figure** **4**a,b). Two approaches were considered for integrating the sensor with the films. The first approach involved dropping the red sensor solution onto the cast bioplastic solution during the wet phase before drying. The second approach involved drying the bioplastic film first and then applying the sensor solution on top, followed by an additional drying step. To determine the most effective design for color application, both methods were tested before and after drying. The best results were achieved with the application method used after drying (Figure 4a‐ii), showing that the color intensity and overall appearance of the smart packaging with the color applied after drying were superior to those with the color applied before drying (Figure 4a‐i). When the colored polymer gel was added to the wet film, it mixed into the film, resulting in a flatter surface with diminished color intensity.

The pH sensitivity of anthocyanins remained intact after casting the polymer gel. Upon contact with water, the plastic became softer and jelly‐like (Figure 4c); however, this effect was less pronounced during vapor exposure, where condensation formed on the film (Figure 4d). Therefore, for food packaging applications, the sensor should not touch the food directly but be exposed only to the vapor released by the food during its decomposition. This reversible pH response allows the sensor to indicate the pH and freshness inside the package even after it has been reopened.

When submerged into a NaOH solution, the color change is clearly visible, transitioning from red to yellow (Figure 4c). However, as the film swelled, it warped and lost stability over ≈10 min. When exposed to vapor instead of liquid, the physical properties of the plastic changed less drastically, with no dilution of the released color observed (Figure 4d). To further evaluate performance, we injected a basic gas (40% NH_3_ in N_2_) to demonstrate the sensor's color change in another pressure and concentration condition. This gas exposure resulted in a more pronounced pH response than direct contact with a solution. The color change was evident, and the physical properties of the film remained stable, aside from some moisture condensation. Importantly, this reaction is reversible; after exposure to pH‐altering vapor, the sensor turns translucent from pH‐neutralization under normal atmospheric conditions and, as illustrated in Figure 4e, it reverts to red upon exposure to an acidic solution or vapor. After the first cycle, no dilution was observed, but slight dilution occurred after multiple cycles. Nevertheless, the sensor retained its functionality and sensitivity after four testing cycles.

To quantify the color changes observed with pH variations, we measured and calculated the ΔE and L/a/b values (Figure 4g–j). The ΔE values for both colored films were relatively similar, showing no significant differences except at pH 6 and 10. In contrast, the L, a, and b values exhibited more variation: The Mono/RRA film appeared lighter in color, while the AM/RRA film had a less pronounced color gradient in the red/green spectrum. Notably, at pH 12, the Mono/RRA film showed a more yellow hue. Both films were suitable for pH‐sensitive applications; however, it is worth noting that the Mono/RRA film became milky at pH 8 and above, which negatively impacts the visual interpretation of the color changes.

### 2.5 Color Response to Shrimp Spoilage

**Figure 5:**
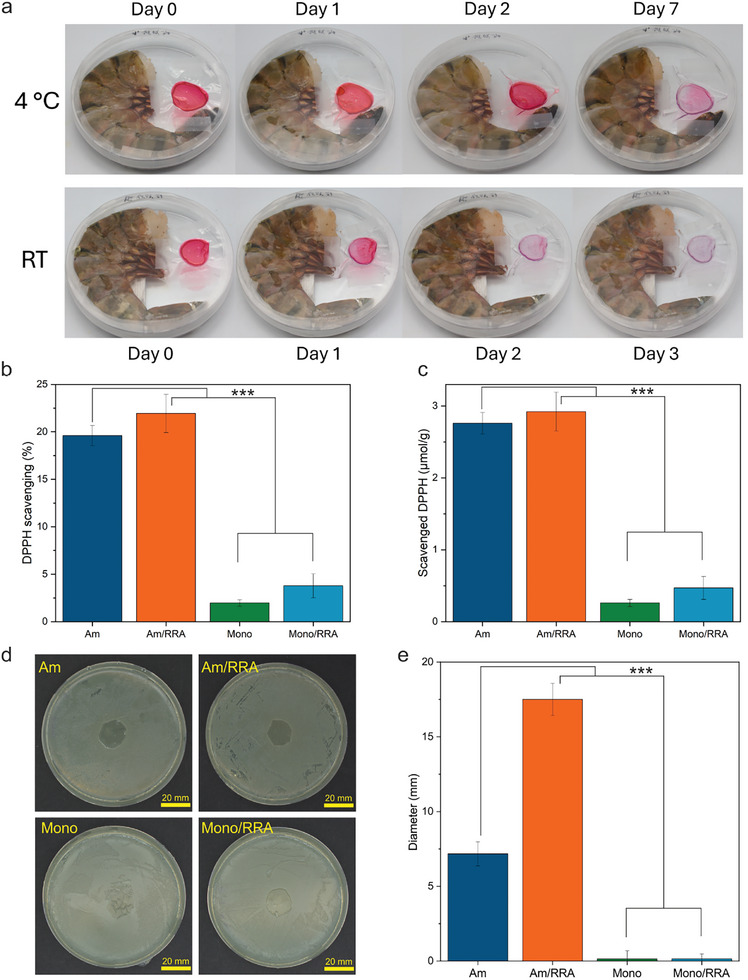
a) Smart sensor changes over time in a Petri dish in the presence of a shrimp at 4 °C and room temperature (RT); Antioxidant assay measured by: b) scavenging rate as the linear change in absorbance before and after the addition of DPPH; and c) scavenged DPPH radicals according to the Beer‐Lamber‐Bouguer Law (Antioxidant test data are represented as means ± D, where *n* = 3, ^***^ *p* < 0.001). D) Antibacterial assay of films in 4.6 × 10^10^ CFU mL^−1^ of E. coli after 96 h (the diameter of the Petri dish is 94 mm). e) diameter in mm after 96 h in 4.6 × 10^10^ CFU mL^−1^ of *E. coli* colony (Antibacterial test data are represented as means ± SD, where *n* = 3, ^***^ *p* < 0.001).

Shrimp is high in free amino acids and unsaturated fatty acids, making it particularly prone to spoilage due to its high moisture content.^[^ ^33^ ^]^ Spoilage occurs primarily through autolytic changes, lipid oxidation, and microbial activity, with the latter posing the greatest food safety risk.^[^ ^34^, ^35^ ^]^ After death, bacteria invade the shrimp tissue, thriving in its neutral pH environment and leading to the accumulation of biogenic amines, particularly histamine, which can cause seafood poisoning.^[^ ^36^ ^]^ Our smart sensor is specifically designed to monitor the volatile nitrogen compounds associated with seafood spoilage.^[^ ^37^ ^]^ As observed in **Figure** **5**a (visually) and Figure S5 (Supporting Information) (quantitatively), the sensor clearly responded to the decomposition process of shrimp stored at room temperature and 4 °C over several days. Within the first 24 h, the sensor's brightness decreased, and by day two, it turned from red into transparent violet/blue, indicating increased total volatile basic nitrogen (TVB‐N) levels. At 4 °C, a response was observed after about seven days. To ensure that these color changes are due to TVB‐N release, the same experiments were done without shrimps (Figure S6, Supporting Information), and no obvious color changes were observed. We further monitored the real‐time degradation of shrimp at RT by measuring pH changes. As shown in Figure S7 (Supporting Information), the pH of the aqueous solution containing shrimp increased from 7.6 to 7.9 over 3 days, indicating progressive sample degradation and demonstrating the sensor's responsiveness to these slight changes in sample basicity.

### 2.6 Antioxidant Activity Assay

Incorporating anthocyanins into food packaging offers significant benefits, primarily through their ability to scavenge free radicals. These free radicals can initiate oxidation reactions, leading to rancidity, off‐flavors, and a reduction in the nutritional value of food products.^[^ ^38^, ^39^ ^]^

Therefore, enhancing packaging materials with antioxidant properties is crucial for extending the shelf life and quality of packaged foods by mitigating oxidative damage.^[^ ^40^, ^41^ ^]^

The antioxidant capacity of the prepared films was evaluated through DPPH (2,2‐diphenyl‐1‐picrylhydrazyl) free radical scavenging activity. The results, shown in Figure 5b,c, illustrate a linear change in absorbance before and after the addition of the antioxidant, reflecting the scavenging rate.^[^ ^42^ ^]^ Figure 5b displays the percentage change, while Figure 5c quantifies the amount of DPPH radicals scavenged in micromoles per gram of film, based on Beer‐Lambert‐Bouguer Law.^[^ ^5^ ^]^ Both figures reveal consistent trends: AM films generally exhibited higher scavenging activity than monomer ones,^[^ ^5^ ^]^ further enhanced by adding anthocyanin dye.

The enhanced antioxidant activity of AM films can be attributed to their unique structural characteristics. The aggregation of AM is stabilized by hydrogen bonding and hydrophobic interactions, resulting in a large surface area with numerous binding sites.^[^ ^43^ ^]^ Antioxidants like anthocyanins effectively interact with these sites, forming hydrogen bonds with amino acid residues in the fibrils and engaging their hydrophobic regions with the fibrils.^[^ ^44^, ^45^ ^]^ This interaction not only stabilizes anthocyanins, protecting them from degradation but also exposes their active sites, facilitating interaction with DPPH radicals.^[^ ^39^ ^]^

In contrast, monomer films, which consist of individual proteins in an unstructured form, do not provide the same level of interaction with antioxidants. They have fewer binding sites than AM, leading to a lower overall scavenging rate, as demonstrated in Figure 5b,c. However, the film prepared with RRA showed slightly higher antioxidant activity, suggesting that even minimal differences in structural organization or the presence of reactive groups can enhance the film's free radical scavenging ability. This underscores the importance of molecular interactions in determining antioxidant efficiency.

### 2.7 Antimicrobial Activity Assay

Incorporating antimicrobial agents such as anthocyanins into food packaging materials provides significant benefits, particularly in inhibiting harmful microorganisms such as *E. coli*. We have further studied the antibacterial properties of our hybrid smart packaging. As observed in Figure 5d, while a distinct inhibition zone was not observed, there was a noticeable difference in the radius from the film's center to the first visible bacterial colony. Visual analysis reveals that films containing AM in a 4.6 × 10^10^ CFU mL^−1^ of *E. coli* exhibited limited bacterial growth, whereas monomer‐based films were completely overgrown. These findings suggest that AM possessed superior antimicrobial properties compared to monomers. The enhanced antibacterial activity of AM can be attributed to their altered charge density and increased hydrophobicity, which facilitate more effective interactions with bacterial cell membranes. Additionally, the structural characteristics of the fibrils enable them to induce significant membrane disruption, leading to pronounced lysis of bacteria.^[^ ^46^ ^]^ Moreover, AM have a higher capacity to incorporate anthocyanins, and adding RRA increases oxidative stress in microorganisms, enhancing the antimicrobial effects against *E. coli* in AM compared to monomers.

To understand the inhibitory effect of anthocyanins, it is essential to consider their bacteriostatic properties.^[^ ^47^ ^]^ *E. coli*, a Gram‐negative bacterium, has an outer membrane rich in lipopolysaccharides (LPS). Anthocyanins can disrupt the bacterial membrane by reducing LPS production, leading to pore formation that increases permeability and compromises membrane integrity. This disruption can result in cell death caused by leakage of intracellular components.^[^ ^46^, ^47^ ^]^ Additionally, flavonoids like anthocyanins may inhibit bacterial growth through mechanisms such as disrupting nucleic acid synthesis or perturbing energy metabolism, although these mechanisms remain incompletely understood.^[^ ^47^, ^48^, ^49^ ^]^

The results, quantified in Figure 5e, demonstrate a consistent trend: larger inhibition diameters were observed when RRA was incorporated into the AM films. However, due to overgrown colonies on both monomer films, no measurable inhibition diameter could be determined in that case, regardless of RRA incorporation.

## 3 Conclusion

In this study, we introduced a sustainable, biodegradable smart packaging solution derived from amyloid fibrils and red radish anthocyanins capable of monitoring seafood spoilage. The films exhibited a strong pH response, transitioning from the flavylium cation (red at pH 2) to various color states as the pH increased, effectively simulating decaying seafood and confirming the packaging's capability to indicate freshness. The food spoiling sensing properties of these films were benchmarked against the real case of shrimp packaging, where at both room temperature and 4 °C, it was possible to follow food alteration with time by simple sensor color changes. The transformation of proteins into AM enhanced the films' properties considerably compared to the monomer‐based control films. Furthermore, the same films also demonstrated high antioxidant and antibacterial activities, highlighting their potential as smart packaging solutions for extending shelf life and improving quality monitoring through environmental responsiveness.

## 4 Experimental Section

### Materials

Whey protein isolate (WPI) was supplied from Fonterra, and PVA, glycerol, ethanol, glacial acetic acid, ammonium hydroxide, and sodium hydroxide were supplied from Sigma–Aldrich. Red radish was bought from a local supermarket (Coop Prix Garantie), a paper filter was bought from Schleicher & Schuell, and a PES membrane of 0.22 µm was obtained from Millipore.

### Methods—Anthocyanin Extraction

A solution‐based method similar to Zhai et al., 2018^[^ ^42^ ^]^ was used. This method followed the steps of peeling the root and drying the skin at low temperatures to avoid thermal degradation of the phenolic compounds, where an oven at 65 °C was utilized for drying. The dried peel was ground into a powder using a coffee mill and extracted in an 85:15 EtOH: HCl (0.1 m) with a solid content of 5%, and this solution was stirred overnight. To remove most particles, a paper filter was used, and the filtered solution was rotary evaporated at 50 °C for 5 h. Afterward, the solution was stored in an aluminum‐wrapped glass bottle at 4 °C. The concentration of RRA solution to acquire the wanted coloration was 1 mg mL^−1^. Additionally, the dry mass was determined by drying in an oven at 120 °C for 5 h and measuring the difference in weight.

### Preparation of AM

For the preparation of the whey AM, 4 g whey protein isolate (WPI) was dissolved in 96 mL Milli‐Q water (Milli‐Q purification system, Millipore), which was pH‐adjusted to a pH‐value of 2 using a 1 m HCl solution.^[^ ^50^ ^]^ The WPI was dissolved under stirring and incubated at 90 °C for 5 h. During this process, the WPI was hydrolyzed and self‐assembled into AM. After forming fibrils, the process was halted by immediately cooling in an ice bath and storing at 4 °C. To check that the AM formation was successful, the cooled‐down solution was looked at under cross‐polarized light to observe AM's birefringence and presence. To determine the impact of anthocyanin on AM formation, 500 mL of 4 wt.% WPI was prepared with and without dye. For the anthocyanin solution, a 1 mg mL^−1^ concentration was used, which was equivalent to 0.1 wt.%.

### Preparation of Biofilm

To prepare the plastic‐forming gel, 100 mL of 4 wt.% AM were mixed with 3 g 146–180 kmol g^−1^ PVA and 3 g of glycerol, which were dissolved at 90 °C for 1 h under stirring.^[^ ^5^ ^]^ This gel‐like solution contained many bubbles, which disturbed the film formation after casting; thus, the solution was centrifuged at 8000 rpm for 3 min to let the bubbles float on top and be removed easily by scooping away the upper layer. Before pouring the gel onto the film coating machine, the TMAXCN Doctor Blade machine was prepared by preheating to 70 °C and vacuum‐attaching a plastic film onto the casting surface to remove the dried film easily. The liquid polymer solution was cast to 1 mm and dried for 45 to 60 min. To compare a monomer‐based film, an identical procedure was conducted for a whey and PVA mixture instead of AM.

To incorporate the anthocyanin into the film, a second layer was added in two ways to analyze which method was better: i) by dripping 1 ml of polymer solution onto the wet film and ii) the pre‐dried film. This gel was analogous to the standard biofilm solution, except it was prepared with AM containing RRA (1 mg mL^−1^). The film was dried for an additional 45 to 60 min. For the smart sensor in the shrimp spoilage experiment, a 10 mg mL^−1^ concentration was chosen to increase the visual difference and depth of color.

### Color Stability of Anthocyanin by Heat

Anthocyanin and other polyphenols were unstable under heat influences.^[^ ^20^ ^]^ This thermal decay follows first‐order reaction kinetics. While water‐based heat degradation was known, the interaction of anthocyanin with AM and its thermal degradation was unknown. Thus, both the absorbance of water and AM‐based anthocyanins were measured at pH 2 by UV–vis. During 3 h, the anthocyanins were exposed to 40 and 90 °C; for every 30 min, one blank and three anthocyanin solutions of each sample were poured into 2 mL cuvettes, and the absorption at λ_max_ (508 nm) was measured.

### Characterization of AM with RRA

To analyze the effect of anthocyanin on the formation of AM from whey, Atomic Force Microscopy (AFM) was used to investigate their morphologies at a nanometer scale.^[^ ^51^ ^]^ The samples were prepared with whey AM and the whey AM‐containing RRA at a concentration of 0.1 wt.% in pH 2 Milli‐Q water. 10 µL aliquots of the solutions were then deposited onto freshly cleaved mica surfaces. Following a 2 min incubation, the samples were gently rinsed with Milli‐Q water and subsequently dried by a gentle flow of compressed air. Finally, the samples were imaged in ambient conditions using tapping mode on a Bruker MultiMode VIII scanning probe microscope.

To evaluate the interaction of anthocyanins with UV light, 3 mL of AM suspensions (4 wt.%) in pH 2 Mili‐Q water were prepared, each with a drop of RRA. The glass vials with these samples were exposed to UV light (365 nm, 3200W AC90V–230V) for 8 h.

The FT‐IR spectra were obtained using a Nicolet iS50 FTIR spectrometer (Thermo Scientific) equipped with an ATR module.

### Characterization of Biofilm—pH‐Response of Smart Sensor to pH‐Changing Solution and Vapors

A pH change in the solution and biofilms was induced by liquid and vaporous pH‐altering substances. For the liquid response, a 0.1 M NaOH solution was used, and for the vapor, a 30% ammonium hydroxide (NH_4_) solution and glacial acetic acid were used to create basic and acidic vapor.^[^ ^52^ ^]^ Ammonium hydroxide simulates volatile nitrogen compounds and acetic acid as an acidic vapor. The solutions covered the pH range of 2 to 12. The biofilms with anthocyanin were submerged on the bottom of Petri dishes, which contained NaOH solution, and photos were taken after a color change had been induced. The gas exposure was tested similarly by using an air‐tight container and adding NH_4_OH and acetic acid, undiluted, a 1:10 water dilution, and a 1:100 water dilution. The films were then put inside the dry container, and photos were taken after the reaction. Additionally, the sensor was tested in 30% NH_3_ in N_2_ to test its monitoring capability in a modified atmosphere.

These color changes can be measured quantitatively with a spectrophotometer CM‐5 [Konica Minolta Sensing Europe B.V.] that can express the perceived color into L*a*b values. These values give information about the brightness (L, 0 = white, 100 = black), redness (a, + = red, – = green), and yellowness (b, + = yellow, – = blue). The color difference can be calculated as ∆E by interpreting color as a vector and by its corresponding length with the Equation (1):

(1) $$\Delta E=\sqrt{{\left(L_{2}-L_{1}\right)}^{2}+{\left(a_{2}-a_{1}\right)}^{2}+{\left(b_{2}-b_{1}\right)}^{2}}$$

To measure the color change on the film, they were quickly submerged in the six solutions representing pH 2 (pure acetic acid), pH 4 (1:10 diluted acetic acid), pH 6 (1:100 acetic acid), pH 8 (1:100 ammonia), pH 10 (1:10 ammonia), and pH 12 (pure ammonia). All samples were measured in a wet state to achieve comparable data.

### Water Vapor Permeability

The water vapor permeability was measured by the change in weight of water‐filled containers covered by the 4 different films; these measurements were taken at the beginning and every few hours.

### Thermogravimetric Analysis

The thermal stability of two film samples was analyzed by heating up to 800 °C in air and N_2_ using a Mettler Toledo TGA/DSC 3+.

### Transmittance of Film

Both RRA and Am/Mono films were analyzed with an Agilent Cary 100 Series UV–vis spectrometer to determine the transmittance to light. The measurements were done by cutting fitting film strips and placing them inside the cuvettes.

### Mechanical Properties of Films

To evaluate the mechanical properties of biofilms, a Z010 (Zwick) textural analyzer was used to measure the forces pulling apart the 3 × 1 cm films to calculate tensile strength, elongation at break, and maximal force at break. From these measurements, the Young's modulus was calculated.

For analysis, the AM film, AM/RRA, Mono, and Mono/RRA were tested.

The calculations are as follows:

(2) $$\text{Tensile strength}=\frac{\text{max.Force}}{\text{Cross}-\text{section area}}$$

where tensile strength is in [Pa], max. Force [N] is the force at breaking, and the cross‐sectional area [m^2^] is the area of the prepared sample (thickness * width).

(3) $$\mathrm{Strain}=\frac{L-L_{0}}{L_{0}}$$

where strain is the percentage of elongation, L [mm] is the current elongation, and L_0_ [mm] is the initial length of the film.

The formula for the Young's modulus is as follows:

(4)

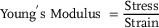

where Youngs's Modulus [Pa] describes the elasticity of material in the linear region.

### Release of Soluble Components

To analyze the behavior of matrix components in contact with water, a release test was conducted.^[^ ^53^ ^]^ The test was executed by adding ≈500 mg AM/RRA and Mono/RRA to 50 mL pH 2 water. The light absorbance of this solution was measured by UV–vis, and 6 measurements were made each of 2 mL cuvettes: the first after 5 min of the films being submerged in water and each following 30 min.

### Color Response on Shrimp

As an application and proof of concept, whole raw shrimp were placed in Petri dishes and sealed with parafilm to keep the vapors inside.^[^ ^53^ ^]^ The experiments were done at room temperature and 4 °C. The AM/RRA smart sensors were attached with tape to the top of the Petri dishes so that they did not contact the subject material. Pictures were taken after starting the experiment and every day after. To observe the real‐time degradation of shrimp at RT by measuring pH changes, a piece of shrimp was placed in a closed container. At designated time intervals, the shrimp was dispersed in Milli‐Q water at a ratio of 0.1 g (shrimp)/mL (water), and the pH of the solution was measured and recorded.

### Antioxidant Activity Assay

The spectral characteristics of anthocyanins provide valuable qualitative and quantitative information regarding their antioxidant activity.^[^ ^54^ ^]^ Spectrophotometry shows particularly great promise due to its simplicity and cost‐effectiveness.^[^ ^55^, ^56^ ^]^ To assess antioxidant activity, a DPPH (2.2‐diphenyl‐1‐picrylhydrazyl) assay was employed following established protocols.^[^ ^57^ ^]^ This assay measures the ability of antioxidants to scavenge free radicals, which was quantified by the decrease in absorbance of the DPPH solution.

The assay procedure involved first dissolving 3.94 mg of DPPH in 100 mL of ethanol (EtOH). To prevent light‐induced degradation of the DPPH solution, the flask was wrapped in aluminum foil.

Four different film types were precisely cut into 20 mg pieces and placed into disposable cuvettes in contact with 2 mL of the DPPH solution. The cuvettes were then kept in the dark for one hour to avoid interference from light.

Absorbance measurements were performed using a UV–vis Spectrophotometer (Agilent Technologies Cary Series) at a wavelength of 517 nm. The absorbance of the control sample, consisting solely of DPPH solution, was recorded immediately before and after the one‐hour incubation in the dark.

The extent of scavenging activity is determined by the decrease in absorbance at 517 nm, which is inversely proportional to the concentration of remaining DPPH radicals in the solution.

The antioxidant activity is quantified using the Equation (5):^[^ ^58^, ^59^ ^]^

(5) $$\mathrm{Antioxidant}\;\mathrm{Activity}\;=(1-\frac{A_{\mathrm{film}\;\mathrm{in}\;\mathrm{DPPH}/\mathrm{EtOH}\;\mathrm{after}\;1\;h}-A_{\mathrm{film}\;\mathrm{in}\;\mathrm{EtOH}\;\mathrm{after}\;1\;h}}{A_{\mathrm{DPPH}/\mathrm{EtOH}\;\mathrm{at}\;0\;h}})\times \;100$$

The calculation of the antioxidant capacity with Equation (6) incorporates the Beer‐Lambert‐Bouguer Law and includes parameters such as the volume of the DPPH solution (*V_DPPH_*), the molar absorptivity (ε), the path length (*l*), and the weight of the film sample (*m*).^[^ ^58^, ^59^ ^]^

(6) $$SC_{\mathrm{DPPH}}=\frac{V_{\mathrm{DPPH}}\times \left(A_{\mathrm{Control}}-A_{\mathrm{Sample}}\right)\times 1000}{\varepsilon \times l\times m}$$

### Antimicrobial Activity Assay

The *Escherichia coli* strain DSM1576 (ATCC8739) was selected for this study.

To determine the colony‐forming units (CFU) of *E. coli*, a bacterial suspension was first prepared by transferring a small amount of *E. coli* from an overnight culture to a sterile tube containing 10 mL of LB nutrient broth, measured using an electronic pipette controller. This suspension was vortexed thoroughly to ensure an even distribution of bacteria and inoculated overnight at 37 °C.

To quantify the initial bacterial suspension, 100 µL of each selected dilution was plated in triplicates onto LB agar plates using a sterile spreader,^[^ ^60^ ^]^ and incubated overnight at 37 °C. After incubation, colonies on each plate were counted using a VWR Colony Counter (Star‐Count STC‐1000). The antimicrobial activity of the films was evaluated using the disk diffusion method. The films were cut into circles with a diameter of 1 cm and placed in sterile, empty petri dishes. The antimicrobial test was performed by spreading 100µl of the bacterial dispersion on LB agar plates, yielding the concentration 4.6 × 10^10^ CFU mL^−1^.

After preparation, the films were placed on the LB agar plates inoculated with bacterial dilution. The plates were incubated at 37 °C for 96 h to observe antimicrobial effects.^[^ ^60^, ^61^ ^]^ The antibacterial nature of anthocyanins was tested by measuring the diameter of the film that was not colonized by bacteria (in mm).^[^ ^39^ ^]^

Quantitative data from all experiments were analyzed using Fiji/ImageJ, an open‐source image analysis software.

### Statistical Analysis

The data were analyzed with R and RStudio (version 4.4.2 R Foundation., Vienna Austria). Results were presented as means ± standard deviation. Significance among means was determined at p < 0.05 by one‐way analysis of variance (ANOVA) with Tukey's test.

## Conflict of Interest

The authors declare no conflict of interest.

## Supporting information



Supporting Information

## Data Availability

The data that support the findings of this study are available on request from the corresponding author. The data are not publicly available due to privacy or ethical restrictions.
