# Supplementary material for: Sustainable Smart Packaging from Protein Nanofibrils
Source: Adv Mater. 2024 Nov 20;37(22):2414658. doi: 10.1002/adma.202414658 (PMC12138864; doi:10.1002/adma.202414658)
Supplement: Supplementary file 1 — Supporting Information [file ADMA-37-2414658-s001.docx]

Supporting Information

**Sustainable Smart Packaging from Protein Nanofibrils**

*Mohammad Peydayesh, Alan Kovacevic, Leah Hoffmann, Felix Donat, Ciatta Wobill, Laura Baraldi, Jiangtao Zhou, Christoph R. Müller, Raffaele Mezzenga^*^*


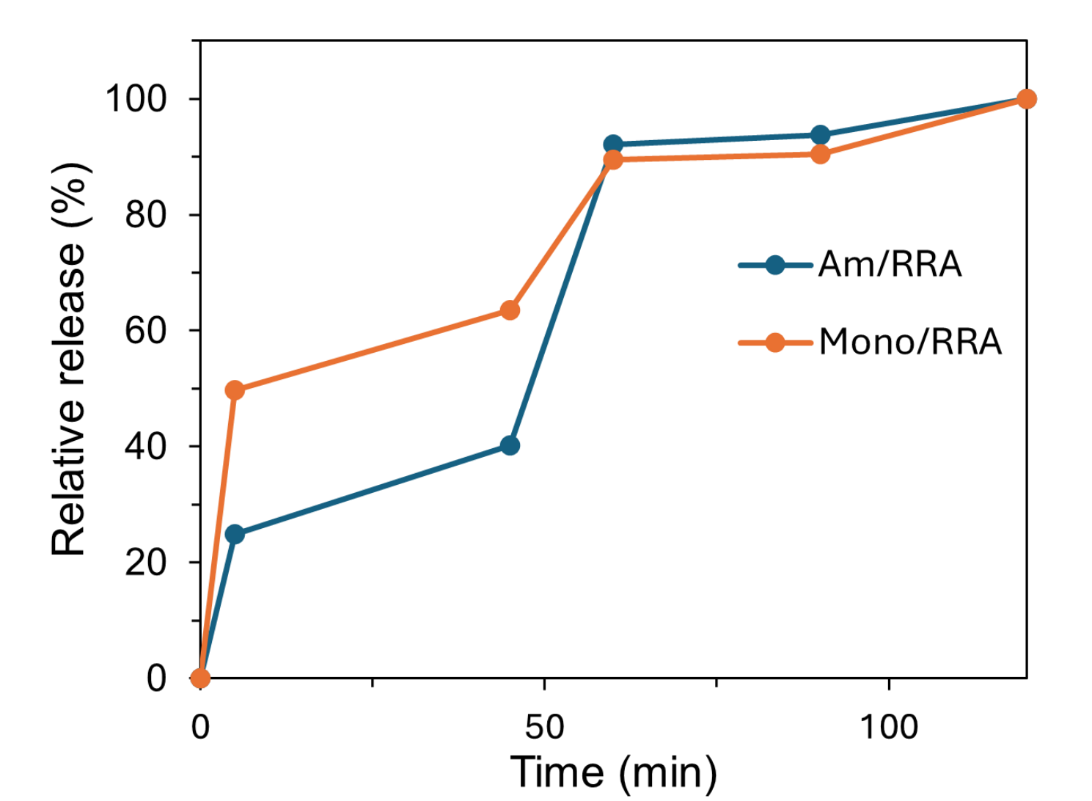


**Figure S1.** Relative release of soluble components from the film.


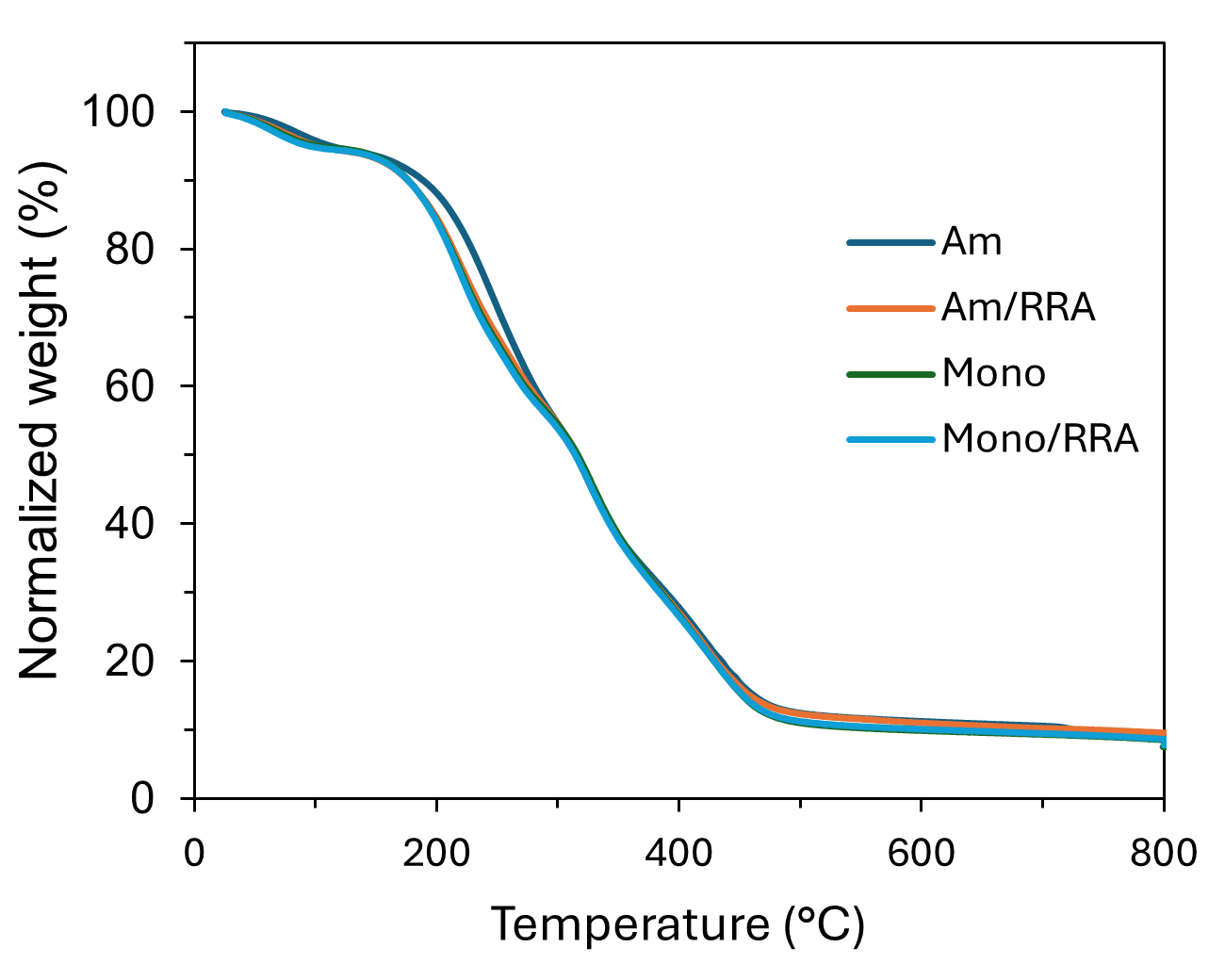


**Figure S2.** Thermal decomposition of biofilms in N_2_ measured in the TGA.


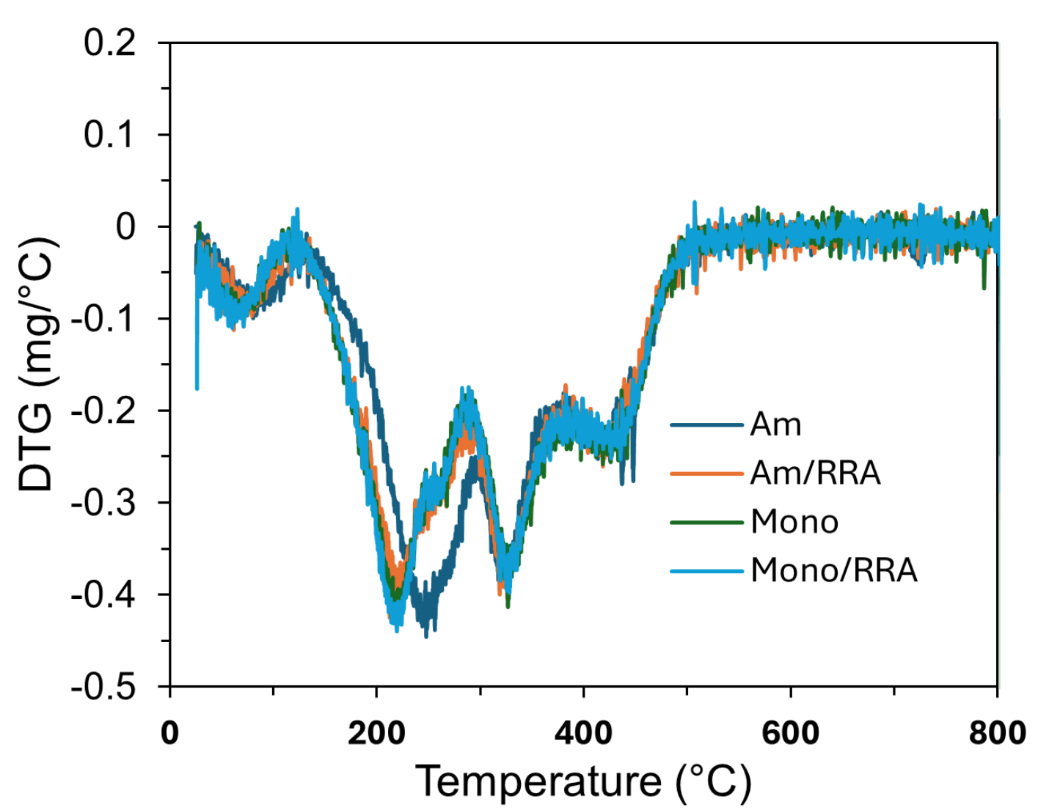


**Figure S3.** Change in normalized sample weight from TGA measurements given in Figure S2.


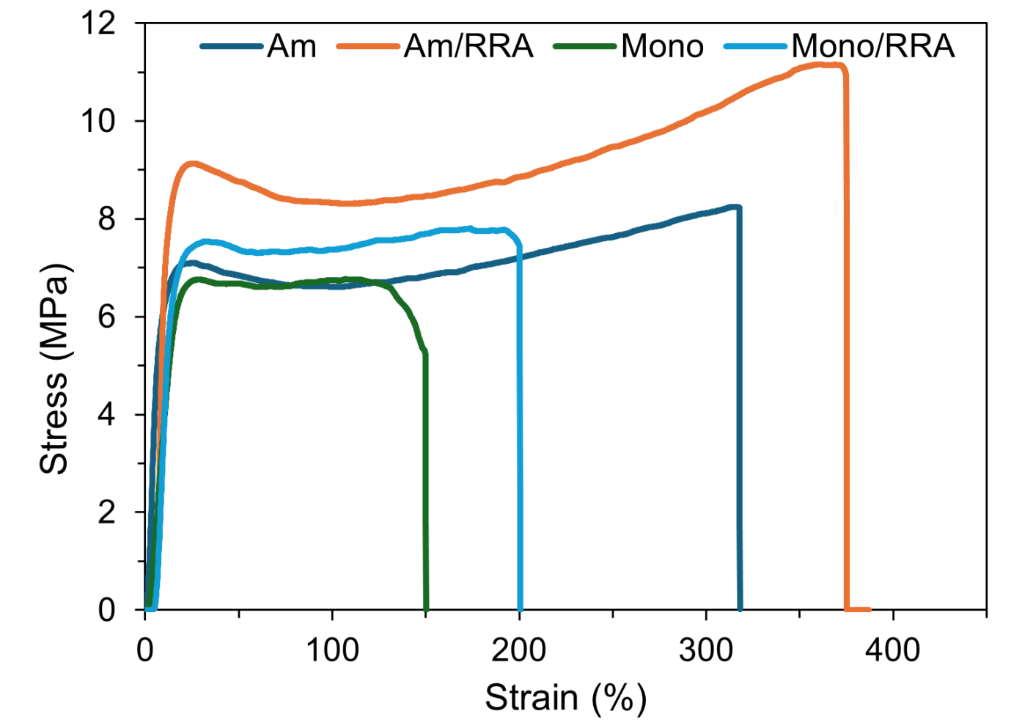


**Figure S4.** Stress-strain curves of samples in MPa vs strain (%).


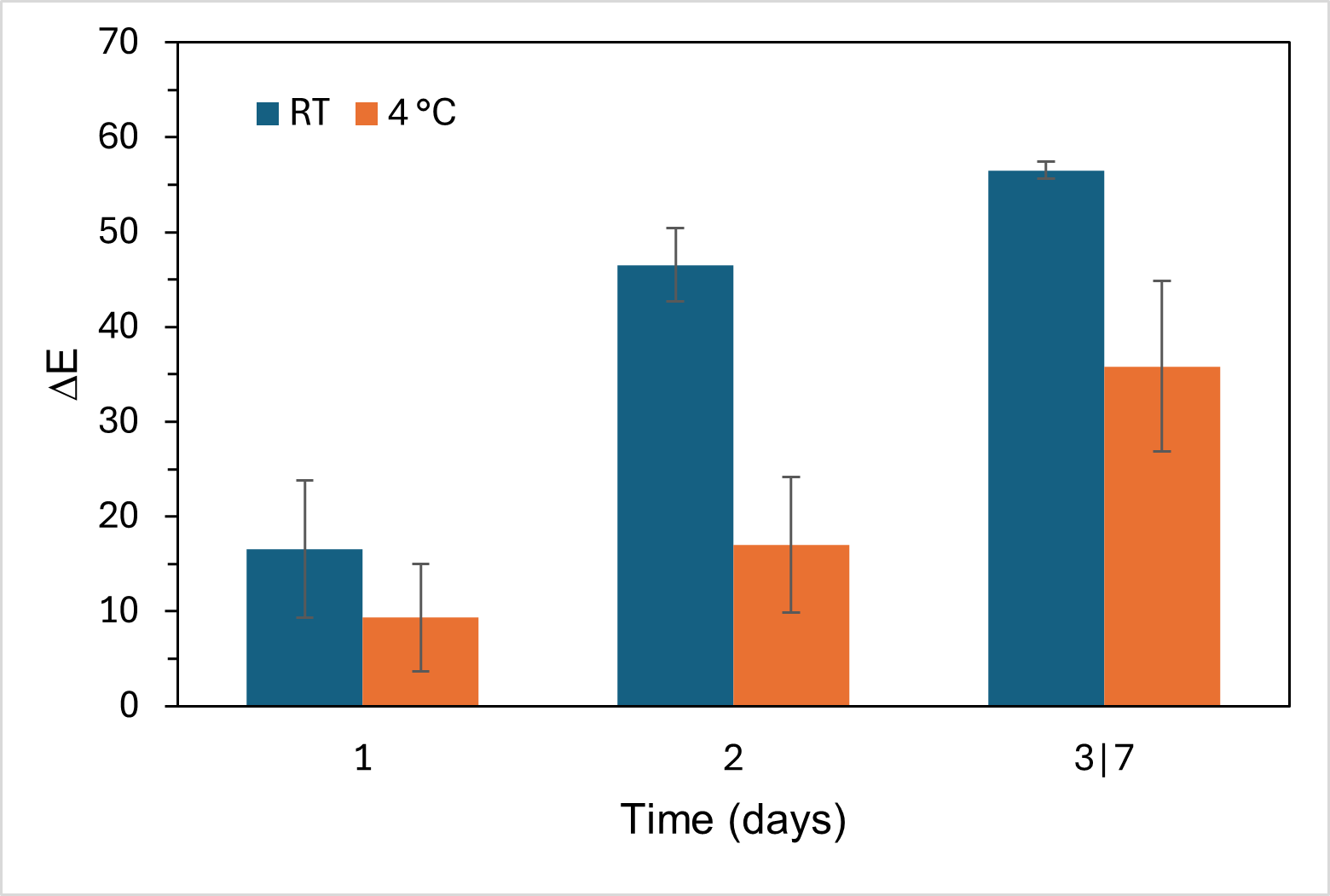


**Figure S5.** Quantified color change of the sensor in the shrimp packaging experiment conducted at room temperature and 4°C (all data are represented as means ± SD, where n = 3).


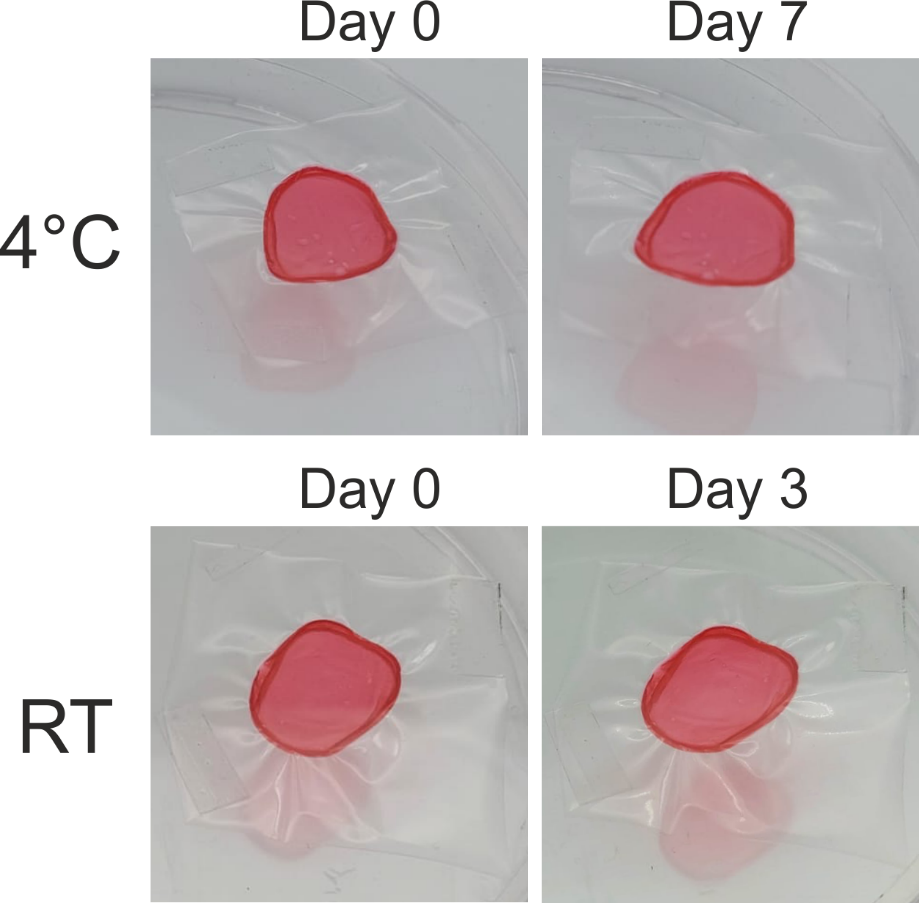


**Figure S6.** Smart sensor changes over time in a Petri dish without a shrimp at 4°C and RT.


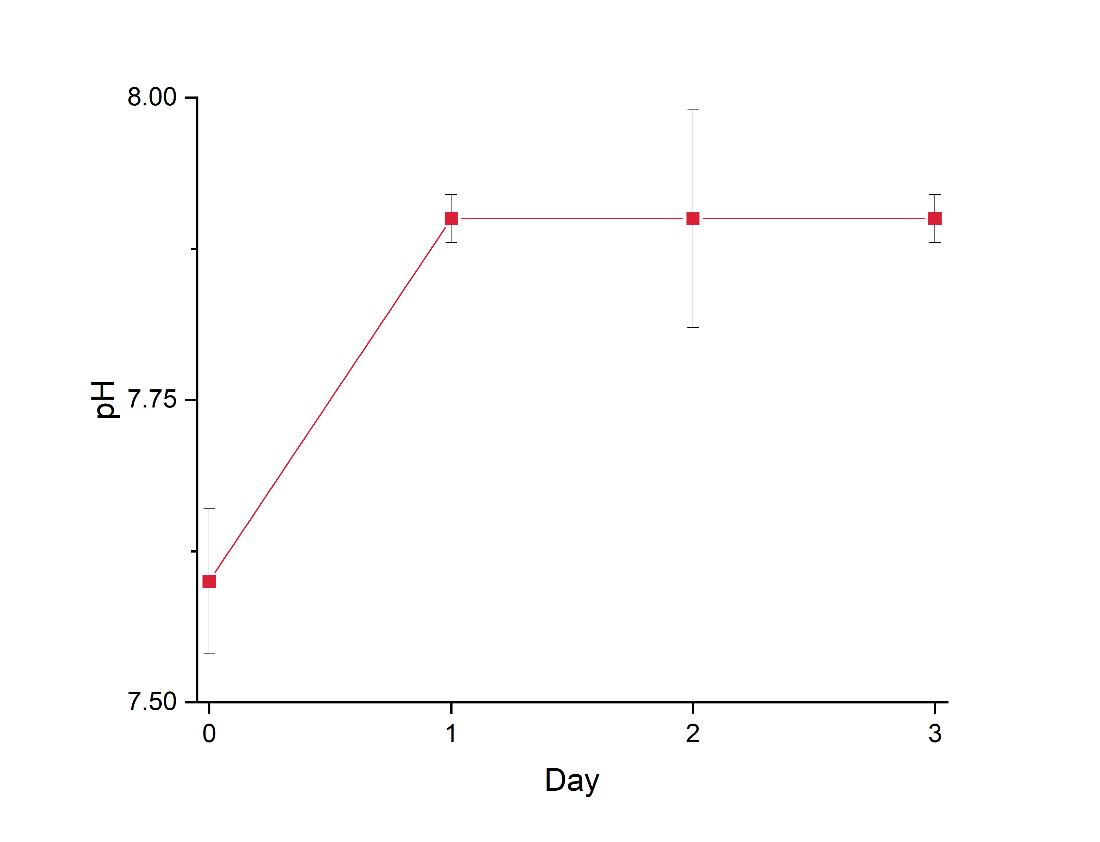


**Figure S7**. pH changes in the aqueous solution containing shrimp aliquots at different time sampling over 3 days at RT (all data are represented as means ± SD, where n = 3).
